# The Application of Induced Pluripotent Stem Cells in Pathogenesis Study and Gene Therapy for Vascular Disorders: Current Progress and Future Challenges

**DOI:** 10.1155/2019/9613258

**Published:** 2019-06-12

**Authors:** Guang-Yin Peng, Yang Lin, Jing-Jing Li, Ying Wang, Hao-Yue Huang, Zhen-Ya Shen

**Affiliations:** Department of Cardiovascular Surgery of the First Affiliated Hospital & Institute for Cardiovascular Science, Soochow University, Suzhou, Jiangsu, China

## Abstract

Vascular disorders are complex diseases with high morbidity and mortality. Among them, the dilated macrovascular diseases (MVD), such as aortic aneurysm and aortic dissection, have presented a huge threat to human health. The pathogenesis of vascular diseases is mostly associated with property alteration of vascular endothelial cells (VECs) and vascular smooth muscle cells (VSMCs). Studies have confirmed that induced pluripotent stem cells (iPSCs) can be proliferated and differentiated into other somatic cells, such as VECs and VSMCs. And patient-specific cells could provide detailed human-associated information in regard to pathogenesis or drug responses. In addition, differentiated ECs from iPSC have been widely used in disease modeling as a cell therapy. In this review, we mainly discussed the application of hiPSCs in investigating the pathological mechanism of different inherited vascular diseases and provide a comprehensive understanding of hiPSCs in the field of clinical diagnosis and gene therapy.

## 1. Background

Vascular diseases include constrictive vascular disease and dilated vascular disease. The former includes vascular pathological stenosis and atherosclerotic plaque obstruction, which are mostly caused by inflammation of the blood vessels. The latter mainly refers to aneurysm and artery dissection [1]. Nowadays, the mechanism research of vascular diseases is mostly focusing on property alteration of VECs and VSMCs. However, these are several limitations: (i) patient vascular specimens are difficult to obtain and (ii) VECs and VSMCs derived from diseased blood vessels are difficult to speculate the complete pathogenesis process; we could not detect the molecular expression levels at different times, observe the morphological and pathological changes at different stages, and study the complete process of cell lesions. As a hot research field today, replacement therapy by stem cells has made great progress, but functional artificial blood vessel is the key to maintaining artificial organ survival. Therefore, the application of iPSC immensely promotes the progress of transplantation therapy [2].

**Figure 1:**
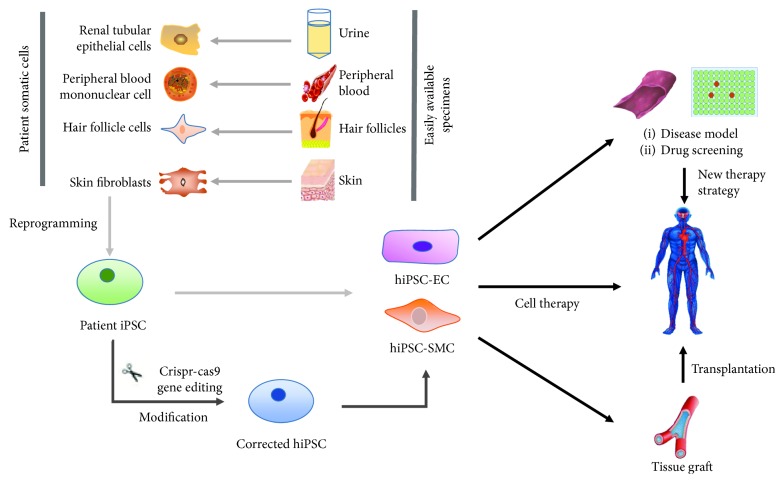
Several types of easily available patient specimens and potential applications of the patient somatic cell-derived hiPSC-EC and hiPSC-SMC. hiPSC derived from the patients' somatic cell such as renal tubular epithelial cell, hair follicle cells, and fibroblast are easily obtained, and the hiPSC derived from the disease-affected cells could be used for disease model and drug screening to study gene function and identify novel pathogenic pathways or therapeutic targets. The hiPSC-derived endothelial cells (hiPSC-EC) or smooth muscle cells (hiPSC-SMC) after gene modification may be an alternative source for cell therapy or tissue engineering repair.

Yamanaka et al. genetically reprogrammed mouse embryonic and adult fibroblasts to a pluripotent state, similar to embryonic stem (ES) cells, by introducing four factors (Oct3/4, Sox2, c-Myc, and Klf4) via viral (retrovirus) transfection. The resulting cells were known as iPSCs. This is the first and most efficient method to generate iPSCs by retroviral introduction of Oct3/4 (also called Pou5f1), Sox2, c-Myc, and Klf4 and subsequent selection for Fbx15 (also called Fbxo15) expression [3]. Because of the significant potential of clinic treatment, researchers attempted to find other methods to avoid the use of integrating viruses. And then, Keisuke et al. repeated the transfection of two expression plasmids, one containing the complementary DNAs (cDNAs) of Oct3/4, Sox2, and Klf4 and the other containing the c-Myc cDNA, into mouse embryonic fibroblasts which resulted in iPSCs without evidence of plasmid integration. Shi et al. identified a small-molecule combination, BIX-01294 and BayK8644, that enables reprogramming of Oct4/Klf4-transduced mouse embryonic fibroblasts, which do not endogenously express the factors essential for reprogramming. With the improvement of the transduction methods, iPSCs could be derived from a wide variety of starting cells, such as renal tubular epithelial cells, peripheral blood mononuclear cells, hair follicle cells, and skin fibroblast; among them, skin fibroblasts are the most common source for their accessibility (can be easily obtained from a skin biopsy) (Figure 1). Other cell types from diverse developmental origins, such as hepatocytes (endoderm origin), circulating T cells (mesoderm), and keratinocytes (ectoderm), have also been successfully reprogrammed into iPSCs even with different efficiency [4]. Because of excellent differentiation potential and no immune rejection, disease models derived from hiPSCs have unique advantages in the study of macrovascular disease [5]. In this review, we mainly introduce the application of hiPSC in elucidating pathophysiological mechanisms of inherited vascular disease and provide a comprehensive understanding of hiPSCs in the field of clinical diagnosis and gene therapy.

## 2. Vascular Cell Models Derived from iPSC

**Figure 2:**
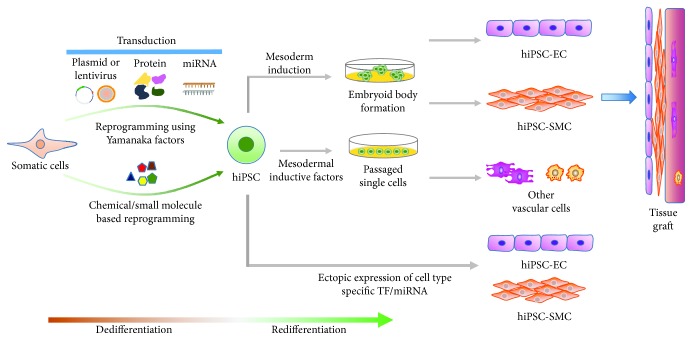
Overview of general strategies for human induced pluripotent stem cell-derived vascular cell generation. Somatic cells can be reprogrammed into human induced pluripotent stem cells (hiPSCs) by transduction of Yamanaka factors or chemical/small molecules, and the hiPSC can be further induced into several functional vascular cell types for vascular injury repair by different methods.

The differentiation methods of hiPSC into EC are summarized into 3 categories: (1) stromal cell coculture; (2) embryoid body (EB) differentiation; and (3) feeder-free monolayer differentiation [6] (Figure 2). Stromal cell coculture was the earliest category used to produce endothelial cells.

It is the method to differentiate EC from iPSCs by coculturing with stromal cells and undirected differentiation and low efficiency, and the produced ECs were also mixed with other cell types. The EB method is also an uncontrolled process, which relies on the spontaneous differentiation of aggregated iPSCs in the context of a 3-dimensional (3D) structure; adding some growth factors such as BMP4, activin A, bFGF, and VEGF could promote this process and increase the differentiation efficiency. Feeder-free monolayer differentiation is to culture a monolayer of iPSC on a matrix-coated culture plate and treat them with different molecules or growth factors to dictate the progressive differentiation from PSC to the mesoderm and finally toward the EC lineage. It is a high-efficiency method that divides PSC into EC lineage from iPSCs. Most studies using this approach can get some of the highest EC yields. By measuring the common features of iPSC-EC, such as the expression of EC surface markers, the formation of capillaries, and the ability to transport LDL, we can give a rough evaluation about the characteristics of iPSC-EC. *In vivo* vascular-forming potential also should be a necessary assay of hiPSC-EC [6]. Researchers have demonstrated that iPSC-ECs increased capillary density and facilitated perfusion in a mouse model of peripheral arterial disease [7].

iPSC-EC-derived vascular models provide a new approach for clinical disease treatment. Patient-specific iPSC-EC can also be used in pathophysiological study or drug discovery. For instance, Tang and his colleagues modeled cadmium-induced endothelial toxicity using hiPSC-derived ECs [8].

The main strategies for deriving SMCs from human iPSCs are divided into two general categories: (1) monolayer method and (2) embryoid body method [9]. In both methods, iPSC was induced to differentiate into the mesoderm, and then, the mesoderm was differentiated into SMC by adding growth factors. The former was to lay a single layer of cells on the surface of Matrigel and treated with specific cytokines, including TGF-*β*1, PDGF-BB [10] or BMP4, FGF-2, and human VEGF [11]. The second method, as an alternative method of the former, aggregates iPSCs, which differentiate into three germline cells in vitro, and obtains SMC cell colonies by negative selection after differentiation of cardiovascular progenitor cells. In addition, Cheung et al. showed the serum-free and chemically defined methods for the production of iPSC-SMC [12].

## 3. iPSC Models for Heritable Large Vessel Diseases (LVD)

### 3.1. Marfan Syndrome (MFS)

Marfan syndrome (MFS) is a heritable systemic connective tissue disease hallmarked by aortic root aneurysms and the subsequent life-threatening dissection and rupture (http://www.omim.org/; OMIM#154700). MFS is always caused by FBN1 mutation and the incidence of MFS is 1 in 3,000 to 5,000 individuals [13]. MFS results in multiple organ lesions, such as skeletal system disorder (arachnodactyly, scoliosis, and pectus excavatum), cardiovascular disease (aortic aneurysm and dissection), and ophthalmopathy (myopia, ectopia lentis) [14], among which a ruptured aortic aneurysm is the most serious. Recently, the emerging of iPSCs has shed light on a new approach in exploring early invasion and disease development of MFS [13, 15–17]. The iPSC-SMCs and iPSC-ECs from MFS patients carrying the FBN1 mutation provide an excellent tool for mechanism investigations, drug screening, and gene editing treatment.

Granata et al. created a vascular model derived from MFS-hiPSCs and recapitulated the pathological process in MFS aortas, including defects in fibrillin-1 assembly, ECM degeneration, TGF-*β* signaling, and SMC contraction and apoptosis. Compared with control hiPSCs (three wild-type hiPSC lines from healthy individuals), fibrillin-1 (coded by FBN1) deposition presented irregular and less enrichment in MFS patients. TGF-*β*1 levels, the density of stress fibers and focal adhesions in MFS-iPSC-MCs, were all significantly higher. Finally, disease phenotype of MFS-iPSC-SMCs (irregular fibrillin-1 accumulation, SMC contraction and apoptosis, etc.) was corrected with CRISPR/Cas9 gene editing, suggesting that this FBN1 mutation C1242Y was causative for MFS. TGF-*β* inhibitor rescued abnormal phenotype of fibrillin-1 accumulation and MMP expression. The noncanonical p38 pathway affected MFS-iPSC-SMC apoptosis, and this pathological process was regulated by Krüppel-like factor 4 (KLF4) [15] (Table 1). This iPSC model declared a novel molecular mechanism of MFS and provided an innovative platform based on human genetic background to test novel treatment targets and drugs.

### 3.2. Loeys-Dietz Syndrome (LDS)

Another heritable large vessel disease similar to MFS is LDS (http://www.omim.org/; OMIM#609192). The two syndromes present some extent of phenotypic overlap for large vessel, skeletal, and skin features. MFS is typically characterized by connective tissue (cardiovascular, ocular, and skeletal) lesions and always caused by heterozygous mutations of *FBN1*. The common cardiovascular phenotypes include thoracic aneurysm and dissection at the sinuses of Valsalva. LDS is an autosomal dominant heritable aortic aneurysm syndrome caused by mutations in *TGFBR1/2* or *SMAD2/3*, all coding for the components in TGF-*β* signaling [25–27]. LDS can be discriminated from MFS by the unique manifestations of hypertelorism, cleft palate or bifid uvular, and widespread aneurysm and arterial tortuosity. LDS cardiovascular phenotype seems more severe than that of MFS [27]. To investigate the specific mechanism of LDS, Hu et al. constructed a hiPSC line from peripheral blood mononuclear cells (PBMC) of a 38-year-old LDS patient with R193W mutation in *TGFBR2* gene [18]. The LDS-iPSCs had a normal karyotype and presented typical human ESC-like colony morphology and proliferative properties. Researchers also performed pluripotency examination. The results suggested that LDS-iPSCs expressed pluripotency markers NANOG, OCT4, SOX2, and TRA-1-60. Teratoma assay further confirmed its pluripotency *in vivo*. In addition, R193W mutation was verified using sequencing. Although they did not analyze the pathophysiological mechanism of LDS in vascular cells and ECM and not confirmed the causative mutation using gene editing and rescue assay, the first LDS-iPSC model has been successfully created for in-depth studies.

### 3.3. iPSC Model for BAV-Related Thoracic Aortic Aneurysm (TAA)

BAV is a congenital defect of the aortic valve (http://www.omim.org/; OMIM#109730). It is characterized by the abnormal fusion of two leaflets among the tricuspid aortic valve [28]. Approximately 50-70% BAV cases will develop to thoracic aortic aneurysm. The incidence is significantly higher than that in tricuspid aortic valve (TAV) individuals [29]. Moreover, a report suggested that patients with BAV displayed a strikingly higher risk for aortic dissection [30]. Whether the aneurysm arise owing to abnormal valve-induced altered hemodynamic force or genetic defect is not clear. Recently, a novel opinion suggested that the ascending aorta, from the aortic root to the aortic arch, is always affected by BAV/TAA, while the descending aorta is not [31]. Embryonic fate-definition studies revealed that the ascending aorta is formed by neural crest- (NC-) derived SMCs. In contrast, the descending aorta is composed of paraxial mesoderm- (PM-) derived SMCs [12, 32]. According to these findings, Jiao et al. created iPSCs from PBMCs of BAV/TAA (Sievers type 1, LCC-RCC fusion) patients. They detected whether the defective differentiation of SMC contributed to aneurysm. Their data suggested that the decreased contractile function of iPSC-NC-SMCs contributed to the development of ascending aortic aneurysm. This contractile defect was not presented in the iPSC-PM- SMCs. This study firstly demonstrated the application of iPSCs in confirming the molecular basis of a BAV/TAA disease process without identifying the underlying genetic defect responsible for cardiovascular abnormality [22].

## 4. iPSC Models for Inherited Small Vessel Diseases (SVD)

### 4.1. iPSC Model for Hutchinson-Gilford Progeria Syndrome (HGPS)

HGPS is a rare human premature aging disease, characterized by the degeneration of VSMCs and premature arteriosclerosis (http://www.omim.org/; OMIM#176670). Progerin, a truncated lamin A, is accumulated mostly in arterial SMCs from HGPS patients, and VSMC degeneration is an essential feature of HGPS-associated arteriosclerosis. To comprehend the underlying molecular basis of HGPS, iPSCs were generated from dermal fibroblasts of HGPS patients. Then, iPSCs were differentiated into neural progenitors, ECs, VSMCs, fibroblasts, and MSCs. Progerin level was notably higher in VSMCs than in neural progenitor cells. This is the first report of an iPSC-based disease model of HGPS [20].

Liu et al. demonstrated that the accumulation of progerin resulted in vascular senescence. The HGPS-iPSC-SMCs they derived from patient skin were expressing characteristic SMC markers such as *α*-SMA and calponin. To mimic the SMC senescence *in vitro*, the differentiated SMCs were serially passaged in common culture. Finally, an increasing number of misshapen nuclei and loss of the heterochromatin marker H3K9Me3 were specifically observed in HGPS-iPSC-SMCs. Moreover, the premature senescence phenotypes of vascular cells, including increased senescence-associated *β*-Gal staining, reduced telomere length, reduced number of Ki67-positive cells, and compromised cell proliferation, were examined in HGPS-iPSC-SMCs [33]. This study provided a unique model system to research human vascular aging pathologies of HGPS.

### 4.2. iPSC Models for Supravalvular Aortic Stenosis (SVAS) and Williams-Beuren Syndrome (WBS)

SVAS is an autosomal dominant disease characterized by abnormal proliferation of VSMCs that can lead to the narrowing of the arterial vessels and a tendency toward sudden cardiac death (http://www.omim.org/; OMIM#185500) [34]. The common causative factors of SVAS are loss-of-function heterozygous mutations of the *ELN* gene that produce haploinsufficiency. Ge et al. reported the development of a human iPSC line from a SVAS patient caused by a 4 bp insert mutation in exon 9 of the *ELN* gene. This report showed that SVAS-iPSC-SMCs had significantly disarranged networks of *α*-SMA filament bundles than WT-iPSC-SMCs. Normal *α*-SMA filament assembly is critical in the contraction of mature SMCs. BrdU analysis declared a significantly higher proliferative and migration potential in SVAS-iPSC-SMCs. They also demonstrated that elevated activity of ERK 1/2 was required for the proliferation of SVAS-iPSC-SMCs [23].

Williams-Beuren syndrome (WBS) is similar to SVAS on the basis of *ELN* mutations and aberrant phenotype of the aorta (http://www.omim.org/; OMIM#194050). WBS is a microdeletion syndrome and has unique phenotypes of craniofacial and neurobehavioral defects compared to SVAS [35]. Kinnear et al. reprogrammed WBS patients' skin fibroblasts to hiPSC and differentiated them into SMCs. WBS-iPSC-SMCs presented reduced differentiated feature, response to vasoactive agonists, angiogenesis, and calcium flux. Administration of elastin-binding protein ligand 2 (EBPL2) partially rescued the SMC morbid phenotype by inhibiting SMC proliferation and facilitating differentiation and angiogenesis. WBS-iPSC-SMCs were used as a tool with an immature proliferative phenotype with decreased contractile properties, thereby recapitulating the human disease phenotype and performing gene therapy of WBS [19].

### 4.3. iPSCs of Pulmonary Arterial Hypertension (PAH)

Heritable PAH (HPAH) is a peripheral artery disease mainly caused by *BMPR2* mutation (http://www.omim.org/; OMIM#178600) [36]. The patients display narrow and stiff pulmonary proximal vessels, predisposing to right heart failure [37, 38]. Endothelial cell dysfunction is essential in the pathophysiology of PAH [39]. Therefore, HPAH-iPSC-ECs were derived from skin fibroblasts of HPAH patients. Functional experiments showed that HPAH-iPSC-ECs had defects in adhesion, tube formation, migration, survival, and BMPR2 signaling when compared with control iPSC-ECs. Transcriptomic analysis declared that high KISS1 and low CES1 levels were associated with reduced migration and survival in patients, respectively. The patient-specific responses to potential HPAH therapies FK506 and elafin were related to antimigratory factor-SLIT3 [40]. Studying gene expression profiling in HPAH-iPSC-ECs resulted in discovering new pathophysiologic mechanisms and screening potential biomarkers associated with drug responsivity.

### 4.4. iPSC of Huntington's Disease (HD)

Huntington disease (HD) is a dominant neurodegenerative disorder which resulted from a CAG repeat expansion in the *HTT* gene, which encodes a mutant HTT (mHTT) protein (http://www.omim.org/; OMIM#143100) [41, 42]. The important characteristics of HD are impaired neurovascular unit (NUV) and blood-brain barrier (BBB) [43, 44]. Brain microvascular endothelial cells (BMECs) are pivotal component in BBB for it protects the brain from toxins and immune attacks [24]. To detect the cell-specific contributions to HD pathogenesis, HD-iPSCs were generated from patient fibroblasts and induced to BMECs. The transcriptome and functional analysis revealed that HD-iPSC-BMECs were intrinsically abnormal in angiogenesis, permeability, barrier properties, and endothelial transcytosis, as well as the signaling critical for these processes. Among them, WNT/*β*-catenin signaling was dysregulated significantly. They further rescued the angiogenic deficits in BMECs via WNT inhibition [24]. This study targeting WNT in HD-iPSC-BMECs manifested novel therapeutic targets in the treatment of HD.

## 5. Gene Therapy in iPSC Models for Vascular Diseases

Because of the lack of stable donor sources, tissue or organ replacement therapy cannot be carried out sufficiently. Therefore, iPSC is a new and better approach to generate tissue and organ. The target organs derived from autologous iPSCs will greatly dismiss immune response during transplantation. The iPSCs corrected by the gene editing technology will repair the pathological lesions and completely cure the disease.

Recently, increasing evidences confirmed that iPSCs modified by CRISPR/Cas9 system could restore the phenotypic abnormality in various heritable vascular diseases, such as MFS [15] and HPAH [21]. Applications of CRISPR/Cas9 gene editing tools to modify the mutation in iPSCs are of great significance for individual therapeutics of genetic vascular diseases. To verify that the phenotypes of MFS C1242Y hiPSC-SMCs are caused solely by C1242Y mutation in the *FBN1* gene, researchers constructed a CRISPR/Cas9 isogenic MFS-hiPSC line [15]. The nucleotide in the mutant allele was substituted with WT nucleotide. These CRISPR-MFS-corrected cells exhibited fibrillin-1 phenotypes consistent with WT cells, comparing to MFS-C1242Y and CRISPR/Cas9-MFS control mutant cells. In CRISPR/Cas9-corrected MFS-iPSC-NC-SMCs, numerous TGF-*β* signaling effectors were inactivated to levels similar to those of WT. Furthermore, TGF-*β* levels in supernatant, MMP10 expression, and matrix degradation in CRISPR MFS-iPSC-NC-SMCs were significantly reduced than before [15]. In conclusion, the combination of iPSC models for vascular diseases and CRISPR/Cas9 gene editing provides a promising platform for the identification of new targets and development of patient-tailored therapies.

## 6. Conclusions

The applications of SMCs or ECs derived from iPSC in understanding the pathogenesis of heritable vascular disease are based on the following merits: (1) in previous researches, animal models cannot fully represent the pathophysiological progression of human diseases. iPSC derived from patient somatic cells is an isogenic disease model better than other models. (2) There are still several drawbacks using ESCs, regarding their origin and generation. In comparison, iPSCs and their target cells after differentiation can be easily obtained and used. (3) The study of vascular diseases cannot be conducted directly, especially for large vessel diseases. The human aorta tissues are difficult to harvest. iPSC can make up for this deficiency. SMCs and ECs derived from iPSCs provide excellent experimental materials in gradual study regarding the pathological basis of cytopathic occurrences. (4) iPSC from patients carries a specific genotype of the individual. It has a unique disease prototype responding specifically to stimulus. Thus, iPSC may theoretically serve as a specific surrogate to test emerging therapies.

In the past decade, many specific hiPSC lines from patients with heritable vascular disease (MFS, LDS, WBS, HGPS, etc.) have been generated, and vascular cells differentiated from these hiPSCs have been applied for pathophysiological mechanism investigation and novel therapeutic discovery. Combined with CRISPR/Cas9 gene editing or other gene therapy intervention, such as WNT inhibitor and EBPL2 peptide administration, the accumulating knowledge on hiPSC-SMC/EC application will benefit the development of novel strategies against heritable vascular disease.

## Figures and Tables

**Table 1:** List of studies using human IPSCs derived from patients for pathogenesis investigation.

Vascular disorders	Gene	Chr, exon, mutation	Pathogenesis-related SMC/EC phenotype and signal changes	iPSC resource	Pluripotency markers (+)	Gene therapy, effect	Author, year	Reference
Marfan syndrome (MFS)	*FBN1*	15, 30, C1242Y	MFS^C1242Y^-iPSC-NC-SMC: proliferative capacity↓, cell death↑, contractility↓, propagating calcium↓, MMP9 expression in stretching↑; MFS^C1242Y^-ECM: fibrillin-1 deposition↓, TGF-*β* signaling↑, collagen I deposition and stiffness↑	Patient dermal fibroblasts	OCT3/4, SOX2, SSEA4, TRA-1-60	CRISPR/Cas9 gene editing, rescues fibrillin-1 phenotype	Granata et al., 2016	[15]
Loeys-Dietz syndrome (LDS)	*TGFBR2*	3, 4, R193W	Not detected	Patient peripheral blood mononuclear cells	OCT4, SOX2, NANOG, TRA-1-60	Not referred	Hu et al., 2017	[18]
Williams-Beuren syndrome (WBS)	*ELN*	7, not referred, 7q11.23 heterozygous deletion	WBS^7q11.23deletion^-iPSC-SMC: immature phenotype↑, proliferation↑, expression of differentiated SMC markers↓, response to vasoactive agonists↓, vascular tuber formation↓, calcium flux↓	Patient skin fibroblasts	NANOG, OCT4, SSEA4	EBPL2 peptide injection, partially rescues WBS cell phenotype	Kinnear et al., 2013	[19]
Hutchinson-Gilford progeria syndrome (HGPS)	*LMNA*	1, 11, G608R	HGPS^G608R^-iPSC-SMC: senescence under hypoxia↑, nuclear dysmorphology↑, DNA damage↑, cell viability↓	Patient skin fibroblasts	OCT4, NANOG, SOX2, SSEA4, TRA-1-80	Reprogramming, low expression of LMNA	Zhang et al., 2011	[20]
Pulmonary artery hypertension (PAH)	*BMPR2*	2, 3, C118W	PAH^C118W^-iPSC-EC: capability of adhesion, survival, migration, and angiogenesis↓, BMPR2 signaling↓	Patient skin fibroblasts	OCT4, KLF4, NANOG, SOX2, TRA-1-81, SSEA4	CRISPR/Cas9 gene editing, rescues PAH cell phenotype	Gu et al., 2017	[21]
Bicuspid aortic valve- (BAV-) related thoracic aortic aneurysm (TAA)	No mutation of *NOTCH1* was found.	Not referred	BAV/TAA-iPSC-SMC: differentiation↓, contraction↓, TGF-*β* signaling↓, mTOR signaling↑	Patient peripheral blood mononuclear cells	OCT4, SOX2, NANOG, SSEA4, TRA-1-60, TRA-1-81	mTOR inhibitor rapamycin, rescues cell phenotype	Jiao et al., 2016	[22]
Supravalvular aortic stenosis (SVAS) syndrome	*ELN*	7, 9, 4 bp insert mutation	SVAS^insert^-iPSC-SMC: property of contractile SMC↓, proliferation↑, migration↑, ERK1/2 activation↑	Patient epicardial coronary artery VSMCs	TRA-1-60, SSEA4, NANOG, OCT4	Elastin, rescues SVAS-SMC phenotype	Ge et al., 2012	[23]
Huntington's disease (HD)	*HTT*	4, not referred, 66 CAG repeats	HD^repeats^-iPSC-BMECs: angiogenic potential↑, migration↑, permeability↑, barrier property↓, endothelial transcytosis↓	Patient skin fibroblasts	OCT4, SOX2, KLF4, L-MYC	Wnt inhibitor rescues angiogenesis defects	Lim et al., 2017	[24]

## Abbreviations

hiPSCs: Human induced pluripotent stem cells
iPSC-LBs: Induced pluripotent stem cell liver buds
ECs: Endothelial cells
SMCs: Smooth muscle cells
VSMCs: Vascular smooth muscle cells
ESCs: Embryonic stem cells
BMECs: Brain microvascular endothelial cells
PBMC: Peripheral blood mononuclear cells
EB: Embryoid body
MFS: Marfan syndrome
HGPS: Hutchinson-Gilford progeria syndrome
WBS: Williams-Beuren syndrome
CFCS: Cardio-facio-cutaneous syndrome
HD: Huntington disease
KLF4: Krüppel-like factor 4
WT: Wild-type
SMA: Smooth muscle actin
SA-*β*-Gal: Senescence-associated *β*-Gal
SVAS: Supravalvular aortic stenosis
ELN: Elastin
PAH: Pulmonary arterial hypertension
CES1: Carboxylesterase 1
SLIT3: Slit guidance ligand 3
KISS1: Kisspeptin 1
TEBVs: Tissue-engineered blood vessels
NF: Nanofibrous
PLLA: Poly(lactic acid).

## Additional Points

*Consent for Publication*. All authors gave their consent for publication. *Highlights*. (1) Human induced pluripotent stem cells have been differentiated into endothelial cells and smooth muscle cells to investigate the mechanism of inherited macrovascular diseases. (2) Human induced pluripotent stem cells are currently applied in providing new research tools and materials for macrovascular diseases and exploring the pathological mechanisms of cardiovascular disease. (3) Functional vascular smooth muscle cells derived from human induced pluripotent stem cells can be used in generating tissue engineering vessel grafts for treatment of vascular diseases.
